# First-Principles Investigation on the Electronic and Mechanical Properties of Cs-Doped CH_3_NH_3_PbI_3_

**DOI:** 10.3390/ma11071141

**Published:** 2018-07-05

**Authors:** Dongyan Liu, Shanshan Li, Fang Bian, Xiangying Meng

**Affiliations:** College of Sciences, Northeastern University, Shenyang 110819, China; dongyanliuNEU@163.com (D.L.); shanshanLiNEU@163.com (S.L.); fangbianNEU@163.com (F.B.)

**Keywords:** perovskite solar cell, DFT calculations, mechanical property, CH_3_NH_3_PbI_3_

## Abstract

Methylammonium lead iodide, CH_3_NH_3_PbI_3_, is currently a front-runner as light absorber in hybrid solar cells. Despite the high conversion efficiency, the stability of CH_3_NH_3_PbI_3_ is still a major obstacle for commercialization application. In this work, the geometry, electronic structure, thermodynamic, and mechanical property of pure and Cs-doped CH_3_NH_3_PbI_3_ have been systematically studied by first-principles calculations within the framework of the density functional theory (DFT). Our studies suggest that the (CH_3_NH_3_)^+^ organic group takes a random orientation in perovskite lattice due to the minor difference of orientation energy. However, the local ordered arrangement of CH_3_NH_3_^+^ is energetic favorable, which causes the formation of electronic dipole domain. The band edge states of pure and Cs-doped CH_3_NH_3_PbI_3_ are determined by (PbI_6_)^−^ group, while A-site (CH_3_NH_3_)^+^ or Cs^+^ influences the structural stability and electronic level through Jahn–Teller effect. It has been demonstrated that a suitable concentration of Cs can enhance both thermodynamic and mechanical stability of CH_3_NH_3_PbI_3_ without deteriorating the conversion efficiency. Accordingly, this work clarifies the nature of electronic and mechanical properties of Cs-doped CH_3_NH_3_PbI_3_, and is conducive to the future design of high efficiency and stable hybrid perovskite photovoltaic materials.

## 1. Introduction

As one of the biggest scientific breakthroughs [1], perovskite solar cell technology has been extensively investigated over the last few years. The conversion efficiency of organic–inorganic lead halide perovskite solar cells has been impressively improved from 3.8% in 2009 to 22.7% recently [2,3,4,5]. In particular, methylammonium lead halide perovskites (ABX_3_; A = (CH_3_NH_3_)^+^; B = Pb^+^; X = Cl, Br, I) are regarded as particularly promising light absorbers because of their outstanding photovoltaic properties. With its continuous increase in conversion efficiency, the stability of CH_3_NH_3_PbI_3_ based solar cells has attracted tremendous attentions for the scale-up of industrial applications [6,7,8]. Enhancing the operational stability of CH_3_NH_3_PbI_3_ without weakening the conversion efficiency is still a major challenge for perovskite-type solar cells [9,10,11].

People attempted to control the composition and proportion of dopants by an alloying method to improve the performance of CH_3_NH_3_PbI_3_-based perovskites. For halogen doping, it has been found that mixing Cl/Br in CH_3_NH_3_PbI_3_ can not only realize the continuous tuning of solar absorption, but also improve the carrier mobility and reduce carrier recombination rates [12,13]. However, due to the thermodynamic instability of the solutions, halogen impurities may cause the segregation of CH_3_NH_3_PbI_3_ into iodide-rich minority and bromide-enriched majority domains under light exposure [14].

Besides the substitution of halogens, B-site doping has also been examined. J. Navas et al. performed experimental and theoretical studies on alloying in Pb^2+^ sites with Sn^2+^, Sr^2+^, Cd^2+^, and Ca^2+^. They pointed out that the Sn^2+^, Sr^2+^, and Cd^2+^ did not modify the phase structure [15]. Y. Ogom et al. proved that the Sn/Pb halide-based perovskite solar cells can harvest the light in the area up to 1060 nm. Nevertheless, the instability caused by the B-site impurities would lead to the reduction of open circuit voltage for CH_3_NH_3_PbI_3_ solar cell materials [16].

Recently, the substitution for A-site organic group in CH_3_NH_3_PbI_3_ has drawn lots of attentions [17,18,19,20]. It has been reported that [CH(NH_2_)_2_]*_x_*(CH_3_NH_3_)_1−*x*_PbI_3_ has favorable performances in terms of structural stability, and the band gap of the A-site solutions can be tuned between 1.48 eV and 1.57 eV [21,22,23]. Yi et al. demonstrated that a mixed A-site cation Cs*_x_*[CH(NH_2_)_2_]_1−*x*_PbI_3_ exemplifies the potential of high efficiency solar cell material [24]. [CH(NH_2_)_2_]_0.85_Cs_0.15_PbI_3_ solution has shown a better performance and device stability than the plain CH(NH_2_)_2_PbI_3_. Compared with the organic (CH_3_NH_3_)^+^ cation, the inorganic Cs^+^ is far less volatile [25,26]. At present, it seems that A-site doping is an effective scheme to improve stability without degrading the light conversion efficiency of CH_3_NH_3_PbI_3_.

In this work, we first carefully studied the of orientation influence of (CH_3_NH_3_)^+^ group on the total energy and band gap in CH_3_NH_3_PbI_3_. After the determination of ground state geometry of pure and Cs-doped CH_3_NH_3_PbI_3_, a comprehensive investigation on the electronic and mechanical properties was performed by first principles calculations. The present study is conducive to the design of perovskite solar cell material with high stability and efficiency.

## 2. Calculation Method and Model

The general chemical formula for perovskites is ABX_3_. In the cubic unit cell of a perovskite, the A-atom sits at cube corner positions (0, 0, 0) in 12-fold coordination, while the B-atom sits at body center position (1/2, 1/2, 1/2) and is surrounded by six X-atoms to form an octahedron group. A-site traditionally is occupied by the inorganic cations, while in the present hybrid perovskite A-site is inhabited by the organic (CH_3_NH_3_)^+^ group. The geometry optimization and the electronic structure calculations of (CH_3_NH_3_)^+^ group are investigated by Gaussian 98 code [27], and B3LYP [28,29] method in connection with the 6-311++G basis set [30,31] is chosen as the exchange correlation potential.

All the bulk geometry optimization, electronic structure, and mechanical property of pure and Cs-doped CH_3_NH_3_PbI_3_ are calculated with ab initio total energy and molecular dynamics program VASP (VASP 5.4.1, Faculty of Physics, University of Vienna, Austria) [32]. Perdew–Burke–Ernzerhof (PBE) pseudopotential with vdw and spin–orbit coupling (SOC) correction is adopted during the calculation [33]. The plane wave kinetic energy cutoff is set to 550 eV and Brillouin-zone integration is performed with a 12 × 12 × 12 Monkhorst–Pack *k*-point mesh. The convergence tolerance for the total energy and Hellmann–Feynman force during the structural relaxation is set to 10^−^^6^ eV and 0.01 eV/Å, respectively.

## 3. Results and Discussion

### 3.1. Intrinsic Properties of CH_3_NH_3_PbI_3_: Structure and Band

**Figure 1:**
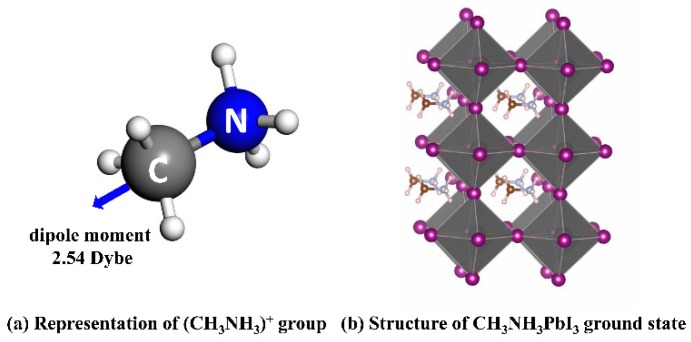
Structure model of (**a**) (CH_3_NH_3_)^+^ group (the arrow represents the direction of electric dipole moment in the group), and (**b**) CH_3_NH_3_PbI_3_ ground state (the PbI_6_ octahedral is rendered).

Different locations of (CH_3_NH_3_)^+^ group in the perovskite lattice would introduce variations in energy and electronic structure due to the intrinsic degree of freedom of the group. Therefore, it is appropriate to start with the study of the nature of (CH_3_NH_3_)^+^. The schematic representation of (CH_3_NH_3_)^+^ group is shown in Figure 1a. N and C atoms are all tetrahedral coordinated, and the length of C-N bond in (CH_3_NH_3_)^+^ group is 1.52 Å, the Van Der Waals volume is 81.67 Å^3^, the density is 0.65 g/cm^3^, and the effective radius of the group is 2.69 Å. The electronic property calculations show that (CH_3_NH_3_)^+^ holds a 2.54 Debye intrinsic dipole moment with the direction from N atom pointing to C atom. As can be seen in the following, the direction of spontaneous polarization of (CH_3_NH_3_)^+^ will influence the stability and bandgap of CH_3_NH_3_PbI_3_.

After the full relaxation of (CH_3_NH_3_)^+^ group, we construct (CH_3_NH_3_)PbI_3_ unit cell based on the cubic APbI_3_ inorganic perovskite frame, in which A-site atom is replaced by the (CH_3_NH_3_)^+^ group. Different from inorganic cation, the organic (CH_3_NH_3_)^+^ group performs a nonspherical shape and can be displayed along different directions in the perovskite lattice [34,35]. In order to determine the energetic favorable configuration, the energy–orientation relationship is studied by fixing the midpoint but rotating the direction of the C-N bond at the center of the cubic cell. Based on the symmetry analysis, there are two independent rotating modes, namely rotating along [110] and [100] directions, for (CH_3_NH_3_)^+^ group and the rotation period of each mode is π/2. The rotating angle is set to zero as the C-N bond lies in the (*ab*) plane.

**Figure 2:**
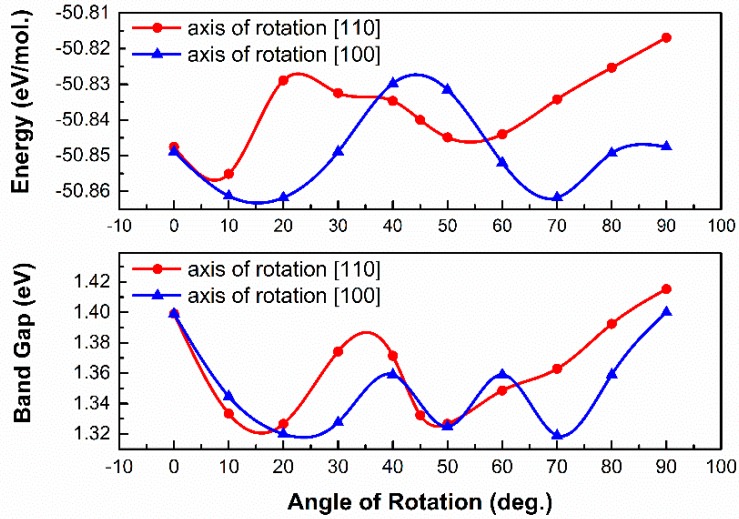
The fluctuation of total energy and bandgap with the orientation of (CH_3_NH_3_)^+^ group in the perovskite lattice.

As shown in Figure 2a, the lowest energies configuration (at 0 K) appears at the position of ±20° when rotating along [100] direction. However, the maximum rotation barrier caused by the orientation is about 40 meV, which is close to the thermal energy perturbation at room temperature (26 meV). As a result, (CH_3_NH_3_)^+^ group will hold a random orientation in the perovskite lattice at RT. Thus, the calculation results explain the disorder arrangement of the (CH_3_NH_3_)^+^group observed in experiments [36,37].

Although the tiny difference in total energy supports the random orientation, we found that the local order location of (CH_3_NH_3_)^+^ group is energetic favorable in configuration. Since G.R. Berdiyorov et al. reported that 48 atoms contained supercell is large enough to negligible the finite size effects [38]. We built supercells containing four unit cells and the orientation of (CH_3_NH_3_)^+^ group in the adjacent cells is arranged as +−+−, ++−−, and ++++. It is found that the all-aligned ++++ mode is the most stable configuration and ~110 meV lower in total energy than the cross +−+− mode. The local ordered location of (CH_3_NH_3_)^+^ is conducive to the establishment of electric dipole domain in the perovskite lattice, which facilitates the separation of photo-generated carriers and leads to the promotion of photoelectric conversion efficiency. This interesting phenomenon has also been investigated by molecular dynamics simulation and experiment [39,40], and the size of the domains is found to be about 100 nm [39,40].

Furthermore, the variation of bandgap with the group orientation is evaluated (As seen in Figure 2b). According to the results, the fluctuation of bandgap caused by the asymmetry of electronic distribution and the electric dipole moment in (CH_3_NH_3_)^+^ group is 0.1 eV. It is interesting that the lowest total energy and the minimum bandgap appear at the same point, namely at the position of ±20° when rotating along [100] direction. Since that the bandgap shrinks with the intensity of electronic hybridization, it is reasonable to deduce that the system has the lowest energy when the electronic bonding is the strongest.

**Figure 3:**
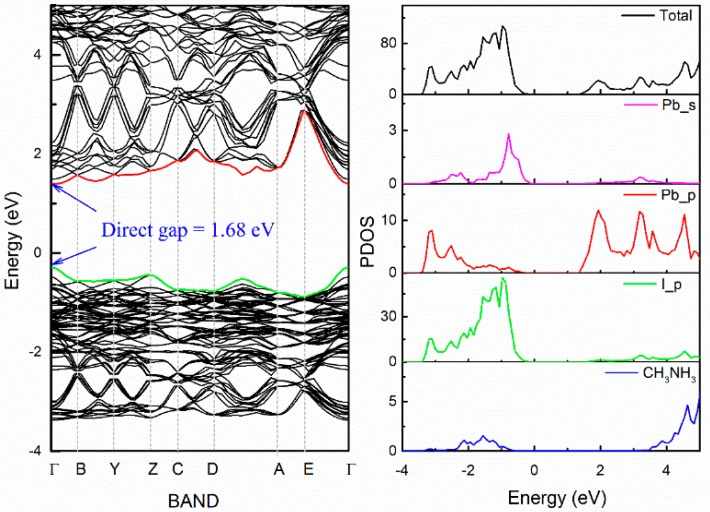
Band level and PDOS of CH_3_NH_3_PbI_3_.

The point with the lowest energy on the energy–orientation curve corresponds to the ground state of CH_3_NH_3_PbI_3_ (Figure 1b). After geometry relaxations, the band structure of ground state in CH_3_NH_3_PbI_3_, drawn between high symmetry points of the Brillouin zone, has been illustrated in Figure 3. CH_3_NH_3_PbI_3_ is found to have a direct bandgap of 1.68 eV at Γ. This result is well consistent with the reported experimental values [41,42,43]. The partial electronic densities of state (PDOS) indicates that the upper valence bands (VB) mainly consist of I-5*p* orbital with weak hybridization to Pb-5*s* state, and the lower conduction bands (CB) are dominated by Pb-6*p* orbital. While the (CH_3_NH_3_)^+^ group has little contribution to the band edge states. The character of band structure in this work is consistent with recent investigations [44,45,46]. As a result, the band gap of CH_3_NH_3_PbI_3_ will be strongly related to the structure of (PbI_6_) inorganic framework. We can thus infer that the influence of (CH_3_NH_3_)^+^ group on the electronic structure belongs to Jahn–Teller effect, that is, the change of electronic structure of the system originates from the distortion of (PbI_6_) octahedral caused by the size and orientation of (CH_3_NH_3_)^+^group, rather than from the direct participation in the electronic structure of (CH_3_NH_3_)^+^. According to our “anion group model” [47], this feature of the electronic structure helps to improve the stability through the element substitution without losing light response and conversion capability.

### 3.2. Cs-Doped CH_3_NH_3_PbI_3_: Stability and Electronic Properties

The structure stability of ABX_3_ perovskites can be estimated by Goldschmidt rule [48], and the tolerance factor *t* of ABX_3_ is determined by the expression

(1) $$t=\frac{R_{A}+R_{X}}{\sqrt{2}(R_{B}+R_{X})}$$

where *R_A_*, *R_B_*, *R_X_* are the respective effective ionic radii of *A*, *B,* and *X* ions. In general, the perovskite structure is considered to be highly stable when the *t*-factor is between the range of 0.90~1.00 [49]. Although the above rule has been developed for the oxide perovskites, but the criterion is still valid for the structural stability analysis of inorganic–organic hybrid halide perovskite materials [50,51].

To determine the range of doping concentration of Cs, the Goldschmidt’s tolerance factors of Cs*_x_*(CH_3_NH_3_)_1−*x*_PbI_3_ (*x* from 0 to 1 at an interval of 0.125) are calculated. In our cases, the effective radii of (CH_3_NH_3_)^+^, Pb^2+^, Cs^+^, and I^−^ in the perovskite-type lattice is 2.69 Å, 1.19 Å, 1.88 Å, and 2.20 Å, respectively. As a result, the *t*-factor is 1.02 for CH_3_NH_3_PbI_3_ and 0.85 for CsPbI_3_. According the criterion of Goldschmidt, the stability of CH_3_NH_3_PbI_3_ and CsPbI_3_ is unsatisfactory. For the Cs doped CH_3_NH_3_PbI_3_, we define the average effective ionic radius of A-site cations as *R_A-eff_* = *x* × *R_Cs_* + (1 − *x*) *R_CNH_*. When the concentration *x* increases from 0 to 1 at an interval of 0.125, the corresponding *t*-factor at each *x* is 1.0, 0.99, 0.96, 0.92, 0.91, 0.89, and 0.87 for the Cs-doped CH_3_NH_3_PbI_3_. Thus, from the view of Goldschmidt rule, the Cs-concentration of 12.5 at.% to 62.5 at.% is more desirable. In experiments, it has been found that Cs-doped CH_3_NH_3_PbI_3_ can be successfully synthesized with a high solid solubility [52].

**Figure 4:**
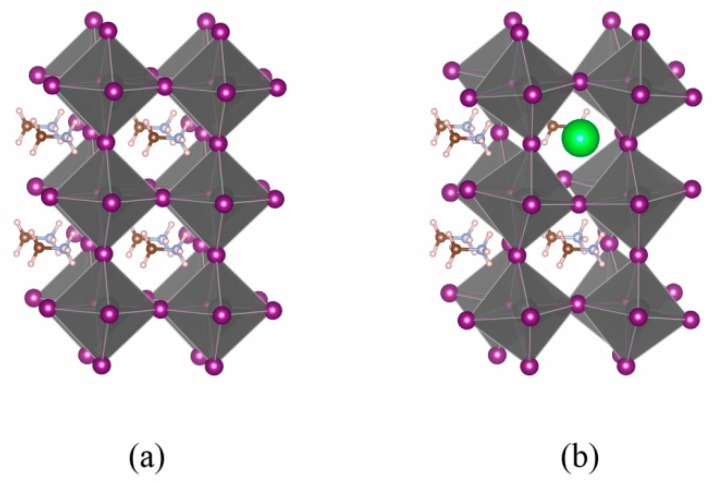
Regular (PbI_6_) octahedral chain in CH_3_NH_3_PbI_3_ (**a**) and distorted chain in 12.5 at.% Cs-doped CH_3_NH_3_PbI_3_ (**b**).

To evaluate the function of Cs-dopant on the structural stability, the 12.5 at.% Cs-doped CH_3_NH_3_PbI_3_ has been modeled by a 2 × 2 × 2 CH_3_NH_3_PbI_3_ supercell with one (CH_3_NH_3_)^+^ group replaced by a Cs atom. In this model we do not consider the disordered orientation of (CH_3_NH_3_)^+^ group. The formation energy of CH_3_NH_3_PbI_3_ and Cs_0.125_(CH_3_NH_3_)_0.875_PbI_3_ are calculated according to the formula

(3) $$E_{formation}=(E_{total}-\sum_{j}n_{j}E_{ion}^{j})/N_{total}$$

where *E_total_* is the total energy of Cs-doped CH_3_NH_3_PbI_3_, $E_{ion}^{j}$ is the energy of constituent elements in their respective elemental state, *n_j_* is the number of various constituent elements, and *N_total_* is the total number of atoms in the supercell. The results show that the formation energy of Cs_0.125_(CH_3_NH_3_)_0.875_PbI_3_ is 0.02 eV/atom lower than that of the pure CH_3_NH_3_PbI_3_, indicating that incorporating Cs into CH_3_NH_3_PbI_3_ lattice is exothermic and helps to stabilize CH_3_NH_3_PbI_3_. More interestingly, it is found that the original regular (PbI_6_) octahedral chain has been distorted in the Cs-doped CH_3_NH_3_PbI_3_, as shown in Figure 4. Thus the symmetry breaking of perfect octahedral chain would also lead to the energy reduction.

**Figure 5:**
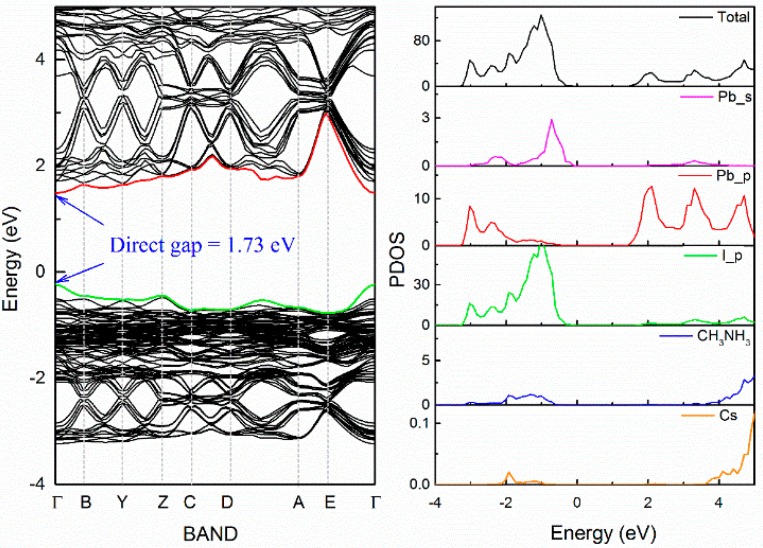
Band level and PDOS of Cs_0.125_(CH_3_NH_3_)_0.875_PbI_3_.

As the incorporation of Cs will induce a 2.75 Debye net electric dipole moment, we introduce an electric dipole correction term in the electronic structure calculation of Cs_0.125_(CH_3_NH_3_)_0.875_PbI_3_. In Figure 5, energy band diagram clearly shows that the bandgap of 12.5 at.% Cs-doped CH_3_NH_3_PbI_3_ is 1.73 eV, being ~3% wider than that of the pure CH_3_NH_3_PbI_3_. It can be seen that the Cs dopant does not introduce new states at the band edges. This result is consistent with the “anoin group model” in that A-site alkali metal or alkaline earth metal cation in perovskites can hardly influence the electronic state near the Fermi level [47], and this is why the alkali metal doping does not cause the deterioration of photovoltaic performance of CH_3_NH_3_PbI_3_. As a result, A-site participation of alkali metal or alkaline earth metal is a promising way to stabilize the hybrid perovskite photovoltaic materials with reliable photovoltaic properties.

### 3.3. Cs-Doped CH_3_NH_3_PbI_3_: Mechanical Properties

It is believed that the mechanical stability of a material is strongly related to its equilibrium elastic modulus and strength performance [53]. The bulk modulus is a measure of resistance to the volume change due to applied pressure, while the shear strength provides information about the resistance of a material against plastic deformation. Thus, the stability of Cs-doped CH_3_NH_3_PbI_3_ should be basically understood by these parameters. To obtain elastic moduli, elastic constants were first calculated from the stress–strain relation, then the Voigt–Reuss–Hill (VRH) approximation was applied to the CH_3_NH_3_PbI_3_ system, and the effective elastic moduli could be approximated by the arithmetic mean of the Voigt and Reuss limits [54].

**Figure 6:**
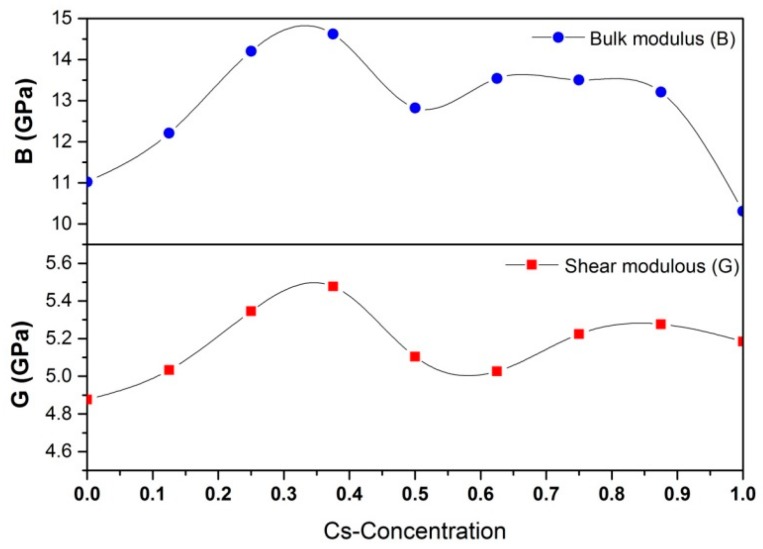
The variation of bulk modulus and shear modulus with Cs-concentration in CH_3_NH_3_PbI_3_.

In Figure 6, our calculations show that the bulk modulus (*B*) of pure CH_3_NH_3_PbI_3_ is 11.0 GPa, and The shear modulus (*G*) is 4.9 GPa, which is comparable to the reported experimental value [55,56]. When the Cs-concentration is below 37.5 at.%, both bulk modulus and shear modulus keep going up and the top value of them is higher than that of pure CH_3_NH_3_PbI_3_ by 3.5 GPa and 0.6 GPa, respectively. When the Cs-concentration increases further, the corresponding elastic modulus decreases with fluctuation. Our calculations suggest that the Cs-concentration should be controlled in the range of 20~35 at.% if we want to achieve the optimal equilibrium mechanical performance. Due to the limitation of solid solubility, the actual Cs-concentration may be lower than this range. However, the elastic moduli still are higher than that of the pure substance, as shown in Figure 6.

According to the work of Pugh, the material is deemed to be ductile if its B/G ratio is greater than the critical value of 1.75 [57]. The B/G ratio of CH_3_NH_3_PbI_3_ is 2.24, while Cs-doped (CH_3_NH_3_)PbI_3_ have higher B/G values indicating that those solutions have a better performance in ductility.

**Figure 7:**
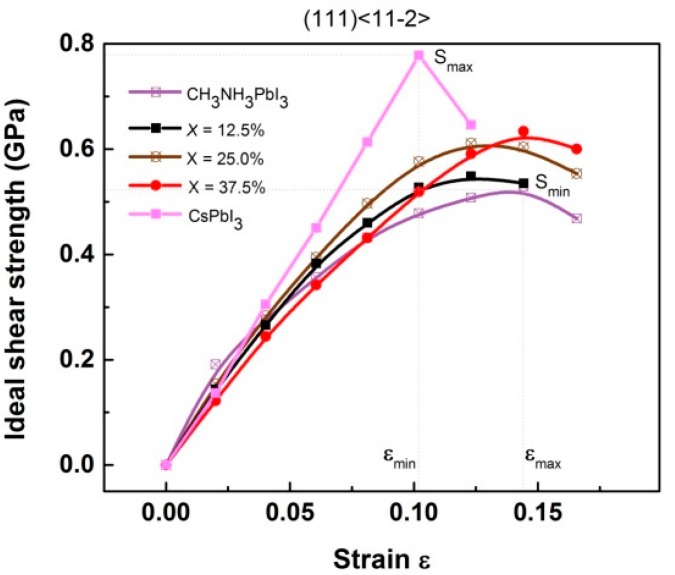
DFT calculated ideal shear stress–strain curves for the (CH_3_NH_3_)PbI_3_ solutions.

The high value of elastic moduli near the equilibrium does not guarantee the high strength. The fracture feature of Cs-doped (CH_3_NH_3_)PbI_3_ should be basically understood from the ideal shear strength obtained far from the equilibrium. The ideal shear stress–strain curves of (CH_3_NH_3_)PbI_3_ solutions in the (111)<11-2> typical slip system are shown in Figure 7.

The strain of deformation-to-failure *ε* indicates the maximum deformation that the material can bear and thus is the measure of the brittleness. It is found that CsPbI_3_ has the highest shear strength (0.78 GPa) but the worst strain capacity, showing a feature of high strength and high brittleness. On the country, (CH_3_NH_3_)PbI_3_ possess the lowest shear strength (0.52 GPa) but the best strain capacity, displaying a feature of low strength and low brittleness. Below 37.5% Cs-concentration, the fracture performance of solutions lies between the boundaries determined by the pure substances CsPbI_3_ and (CH_3_NH_3_)PbI_3_, that is, the ideal shear strength increases but the maximum bearing deformation decreases. It should be noted that the current strength calculations are strictly related to the defect free lattice, and the obtained ideal might overestimate real shear strengths. Along with the analysis on the equilibrium elastic moduli, we can conclude that below 37.5% Cs-concentration (CH_3_NH_3_)PbI_3_ will possess a desirable performance in mechanical properties including stability, hardness, strength, and ductility.

## 4. Conclusions

Aiming at developing new efficient and stable perovskite solar cell materials, we report a comprehensive theoretical investigation on the geometry, electronic structure, and mechanical property of pure and A-site Cs-doped CH_3_NH_3_PbI_3_. The main conclusions are drawn as follows:

(1) The difference in orientation energy of (CH_3_NH_3_)^+^ is comparable to the thermal power at room temperature, which causes a random orientation of (CH_3_NH_3_)^+^ group in the perovskite lattice.
(2) The local ordered arrangement of (CH_3_NH_3_)^+^ is energetic favorable that facilitates the formation of the electronic dipole domain, which helps to improve the separation and lifetime of photo-generated carriers.
(3) The band edge states are dominated by (PbI_6_) anion group in CH_3_NH_3_PbI_3_. A-site (CH_3_NH_3_)^+^ or Cs^+^ does not directly participate in the construction of the band edge states, but indirectly influences the structural stability and electronic level through Jahn–Teller effect.
(4) It has been demonstrated that the suitable concentration of Cs can enhance both thermodynamic and mechanical stability of CH_3_NH_3_PbI_3_ without deteriorating the conversion efficiency.
(5) Goldschmidt’s tolerance factor suggests that the Cs-concentration should be less than 62.5 at.%, while mechanical performance indicates that the optimal Cs-concentration should be less than 37.5%. Below this mark, the mechanical properties including stability, hardness, strength, and ductility will continuously rise with the Cs-concentration.

The adopted research methods, mechanism cognitions and obtained conclusion in the work might help contributing to the future development of efficient and stable hybrid perovskite solar cell materials.
